# Laparoscopic Approach for Gallstone Ileus in Geriatric Patients

**DOI:** 10.7759/cureus.8642

**Published:** 2020-06-15

**Authors:** Anupam K Gupta, Oscar A Vazquez, Jose F Yeguez, Bruce Brenner

**Affiliations:** 1 Minimally Invasive Surgery, University of Miami Hospital, Miami, USA; 2 Surgery, Charles E. Schmidt College of Medicine, Florida Atlantic University, Boca Raton, USA; 3 Surgery, Boca Raton Regional Hospital/Charles E. Schmidt College of Medicine, Florida Atlantic University, Boca Raton, USA

**Keywords:** gallstone ileus, gallstones, bouveret's syndrome, laparoscopy, geriatrics, surgery

## Abstract

We report two patients who presented with small bowel obstruction secondary to gallstones in the ileum. Both patients were geriatric women with multiple comorbidities. The first patient was a 73-year-old woman who presented with a gallstone eroding and obstructing the duodenum (Bouveret's syndrome) secondary to gallbladder cancer with diffuse metastatic spread to the liver. The stone was disimpacted endoscopically using lithotripsy. The patient presented two days later after the stone had migrated downstream into the small bowel causing obstruction requiring surgical intervention. Second patient was an 81-year-old woman who presented with small bowel obstruction caused by a gallbladder stone impacted in the distal ileum. Both patients were managed laparoscopically with a mini laparotomy to extract the affected segment of bowel loop via small incision on the anterior abdominal wall at the port site with enterolithotomy. Both patients were discharged by postoperative day four with no complications. We conclude that, in elderly patients with multiple comorbidities presenting with gallstone ileus, laparoscopic approach provides early recovery with minimal pain.

## Introduction

Gallstone ileus is a rare complication of cholelithiasis which is defined as a mechanical intestinal obstruction caused by impaction of one or more gallstones in the ileum after being passed through a biliary-enteric fistula [1]. Its manifestation is usually preceded by pericholecystic inflammation after cholecystitis which leads to the development of adhesions between the biliary and enteric tracts. Pressure necrosis by the gallstone against the biliary wall then causes erosion and fistula formation [2]. Additionally, cases of gallstone ileus have occurred after endoscopic sphincterotomy where the stone is presumed to have passed into the small bowel through the sphincterotomy and to have been large enough to cause obstruction [3]. Gallstone ileus is an unusual etiology for small bowel obstruction accounting for less than 1% of patients who present with mechanical small bowel obstruction; about 0.01% in a recent large study [4]. This condition develops in 0.3%-0.5% of patients with cholelithiasis and has also been shown to disproportionately affect elderly women [5,6]. One of the patients presented with Bouveret’s syndrome, the most infrequent variant of gallstone ileus, with a little over 300 cases in literature since its first description in 1654 through 2008 [7]. Bouveret’s syndrome results from a gallstone entering the intestinal lumen through a fistula between the gallbladder and a portion of the stomach or intestine. This leads to obstruction at the gastric outlet and, occasionally, in other portions of the intestine as well [8].

## Case presentation

**Figure 1:**
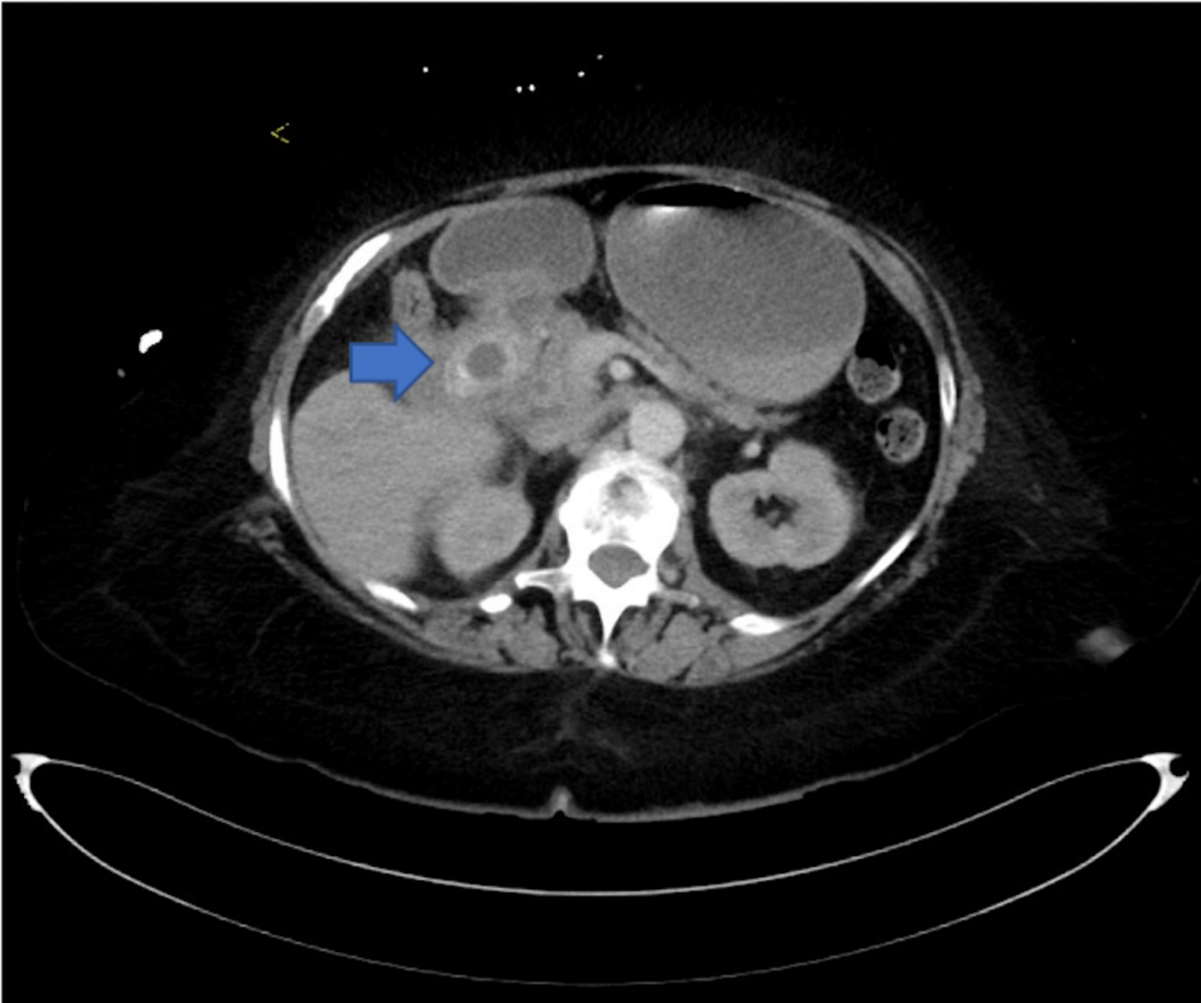
Gallstone impacted in the duodenum (Bouveret syndrome).

**Figure 2:**
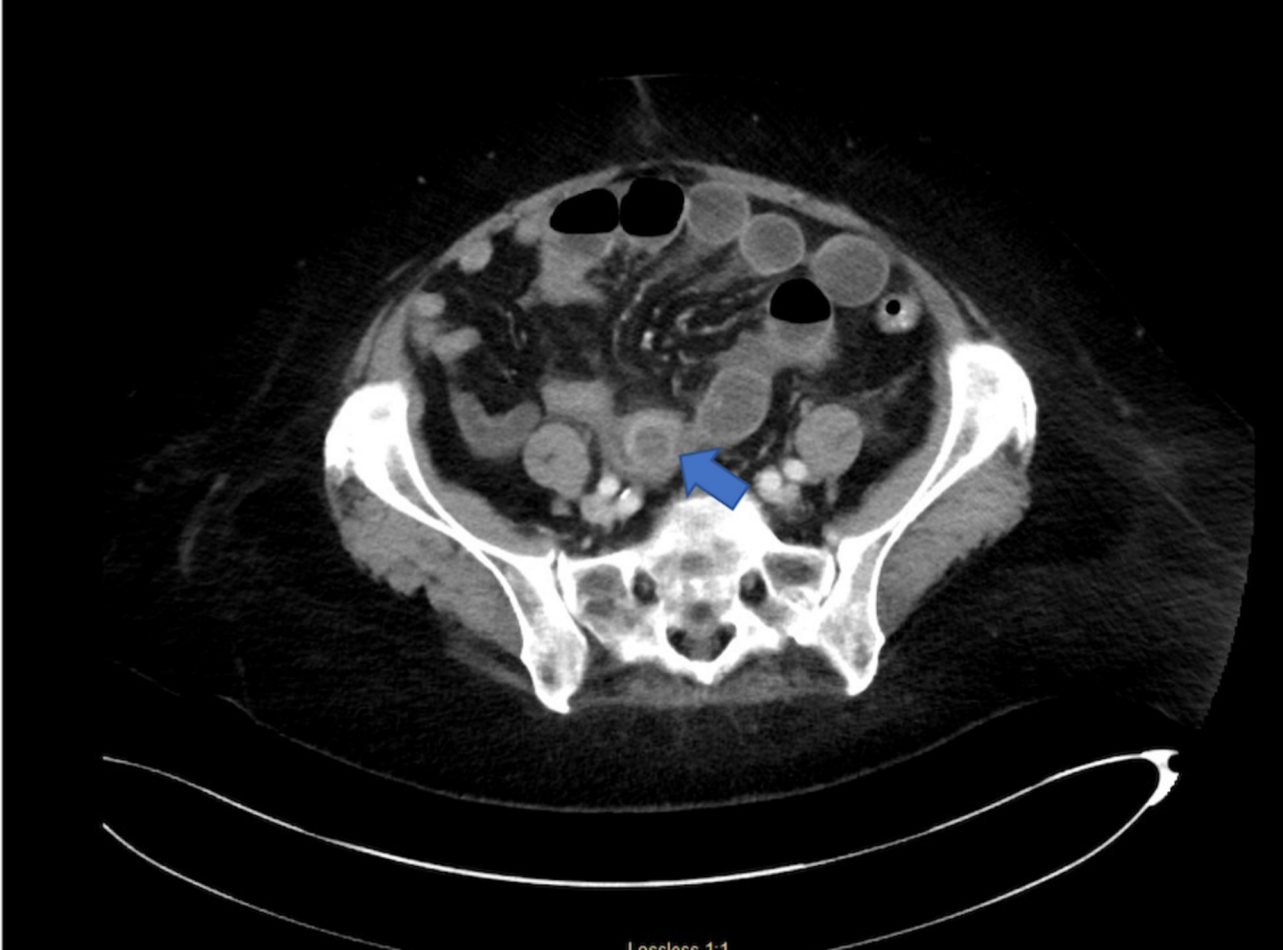
Impacted gallstone in distal ileum.

**Figure 3:**
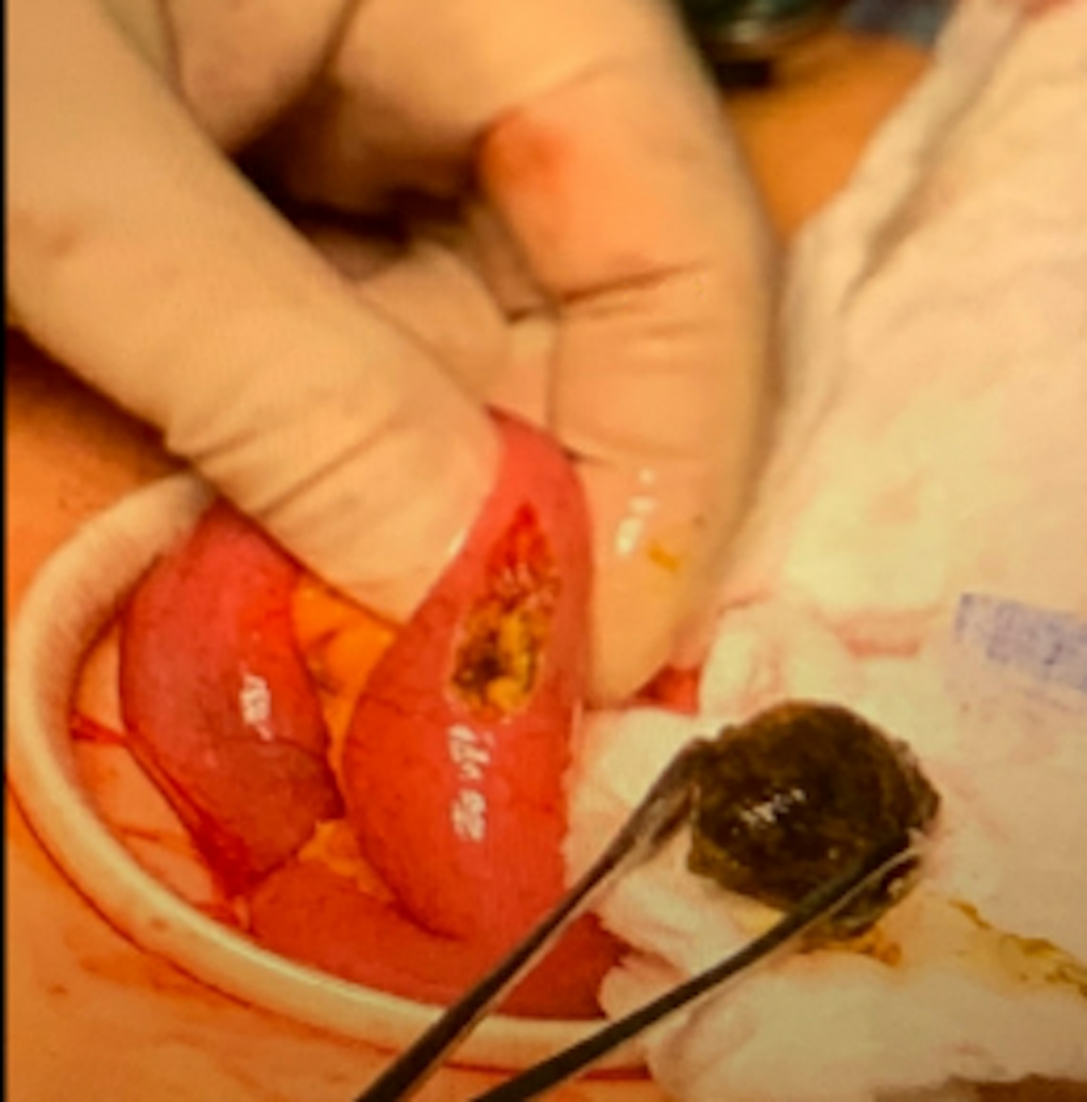
Enterotomy to extract the gallstone.

First case is a 73-year-old, weak, and debilitated woman with a Karnofsky index of 40 (needing special care for personal needs) who was known to have gallbladder cancer with multiple metastatic nodules to liver, anemia, prior stroke with residual weakness, and hypothyroidism. She presented to the emergency room with nausea, vomiting, and abdominal discomfort. Computed tomography (CT) imaging revealed impacted stone in the duodenum causing gastric outlet obstruction (Figure 1). She underwent endoscopic hydraulic lithotripsy to disimpact the obstruction and help in relief of her symptoms. She subsequently presented to the emergency room two days later with complaints of nausea, vomiting, abdominal pain, and obstipation. Her vital signs showed a temperature of 36.8°C oral, heart rate of 96 beats per minute, and oxygen saturation of 97% on room air. Her blood work at the time of arrival was suggestive of hemoconcentration secondary to dehydration due to extensive vomiting. Her abdominal examination was positive for bloating. She was evaluated by CT of the abdomen which revealed presence of an impacted stone in distal small bowel with dilated loops proximal to it (Figure 2). She was emergently taken to the operating room for laparoscopic enterolithotomy. Three ports were used, one video port at the supraumbilical position, and two additional 5-mm ports in left upper and left lower quadrant. The decompressed loop of ileum was identified and run backwards to identify the transition point. The affected segment of bowel loop was grasped and brought out through the supraumbilical port site by making of 5-cm mini laparotomy. Enterotomy and stone extraction was performed (Figure 3). Postoperative course was uneventful, the patient was started on a diet on postoperative day one and was discharged on postoperative day 4.

**Figure 4:**
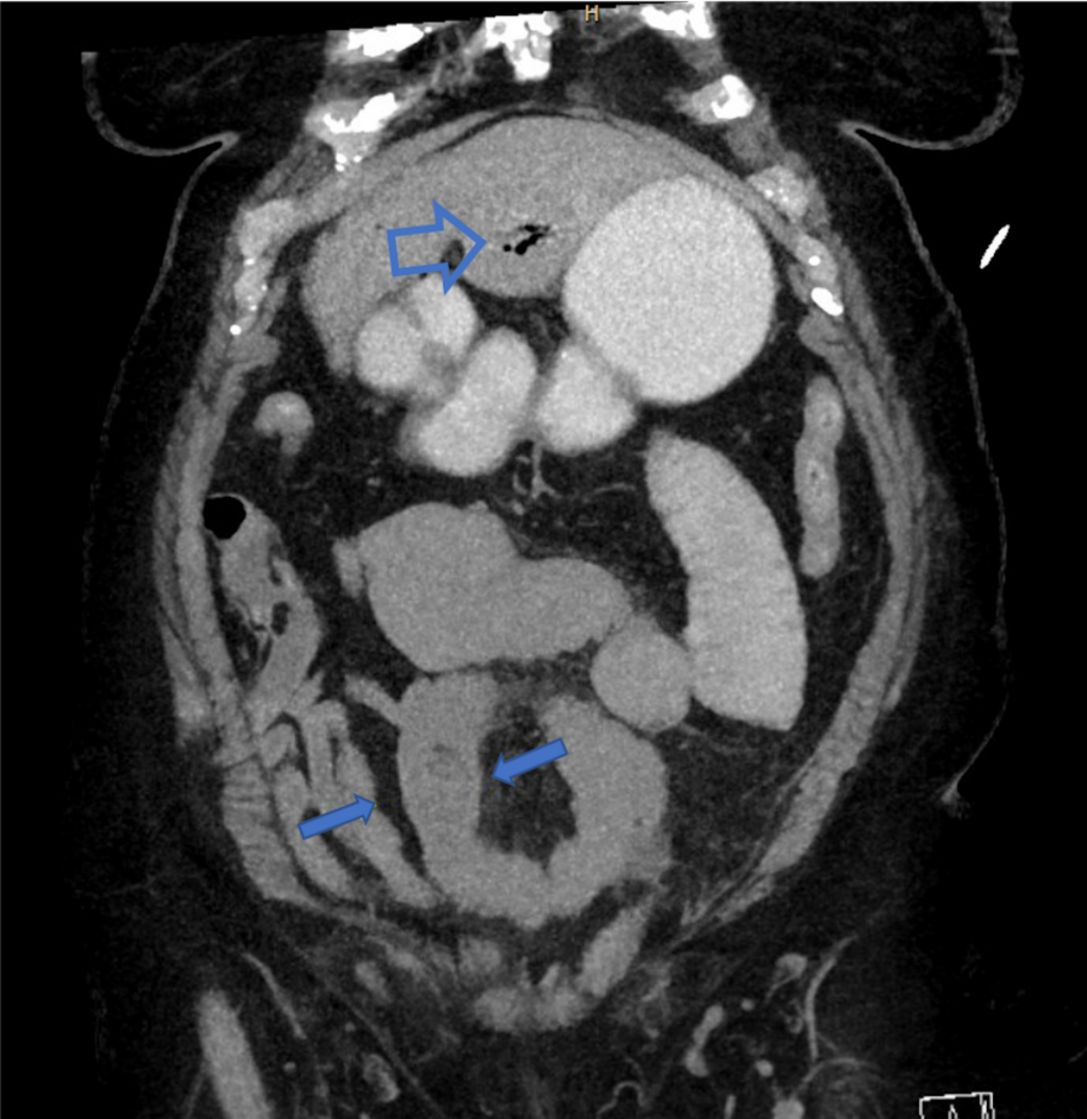
Pneumobilia (top hollow arrow) with gallbladder stone in distal ileum with dilated loops of small bowel (bottom solid arrows).

**Figure 5:**
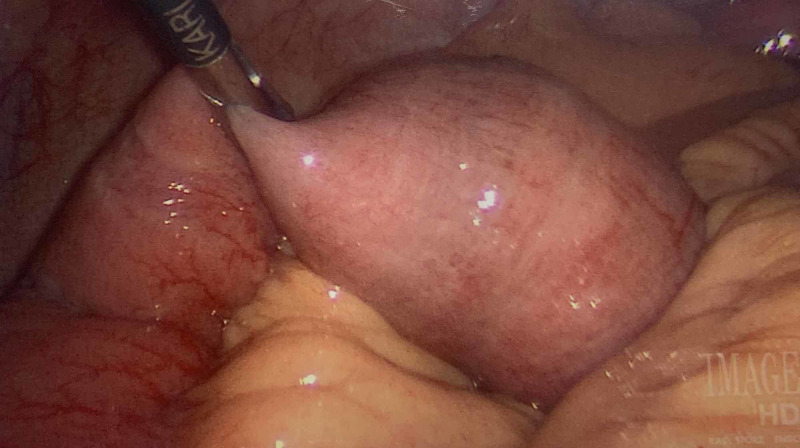
Laparoscopic view of gallstone in the small bowel

**Figure 6:**
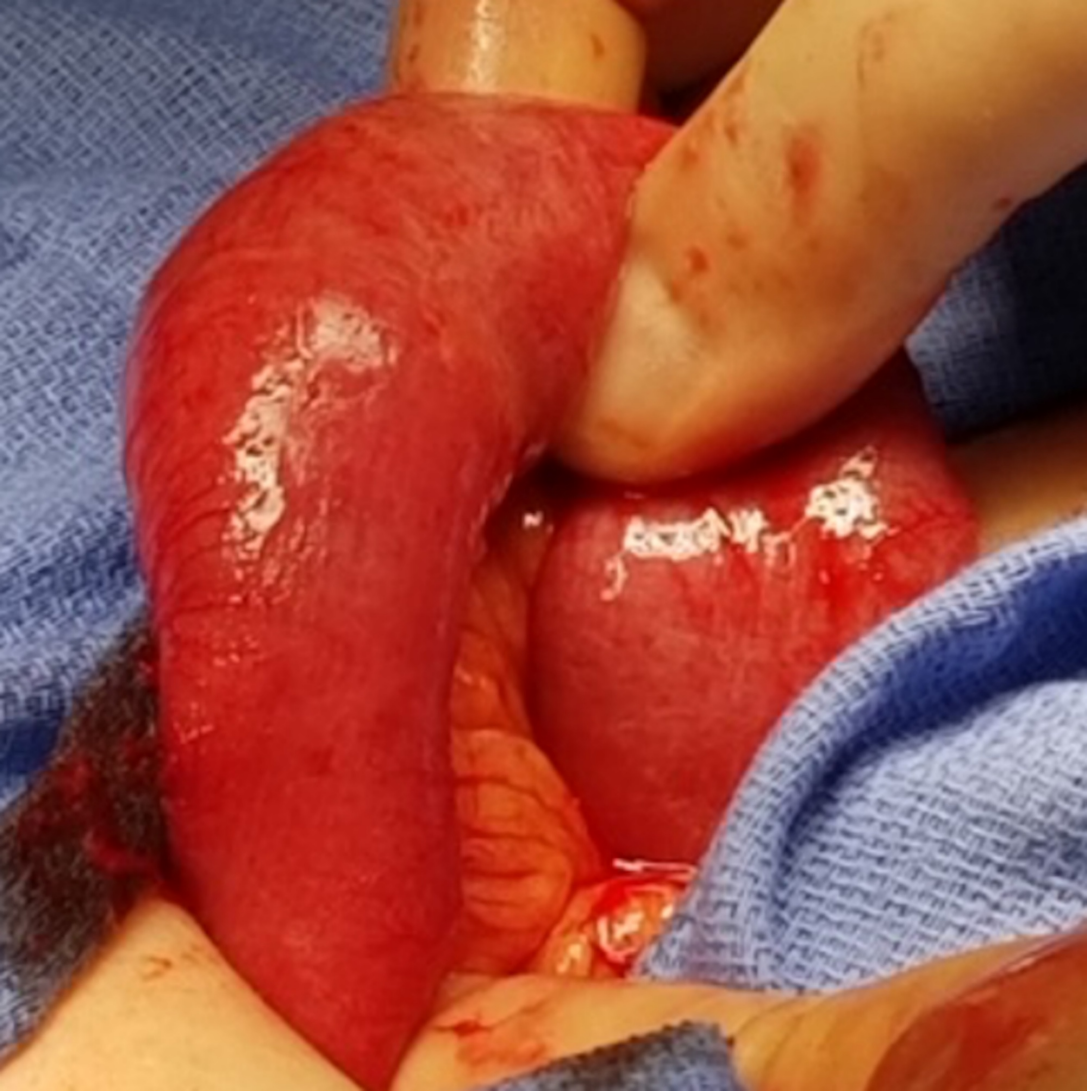
Segment of bowel brought out to perform enterolithotomy

**Figure 7:**
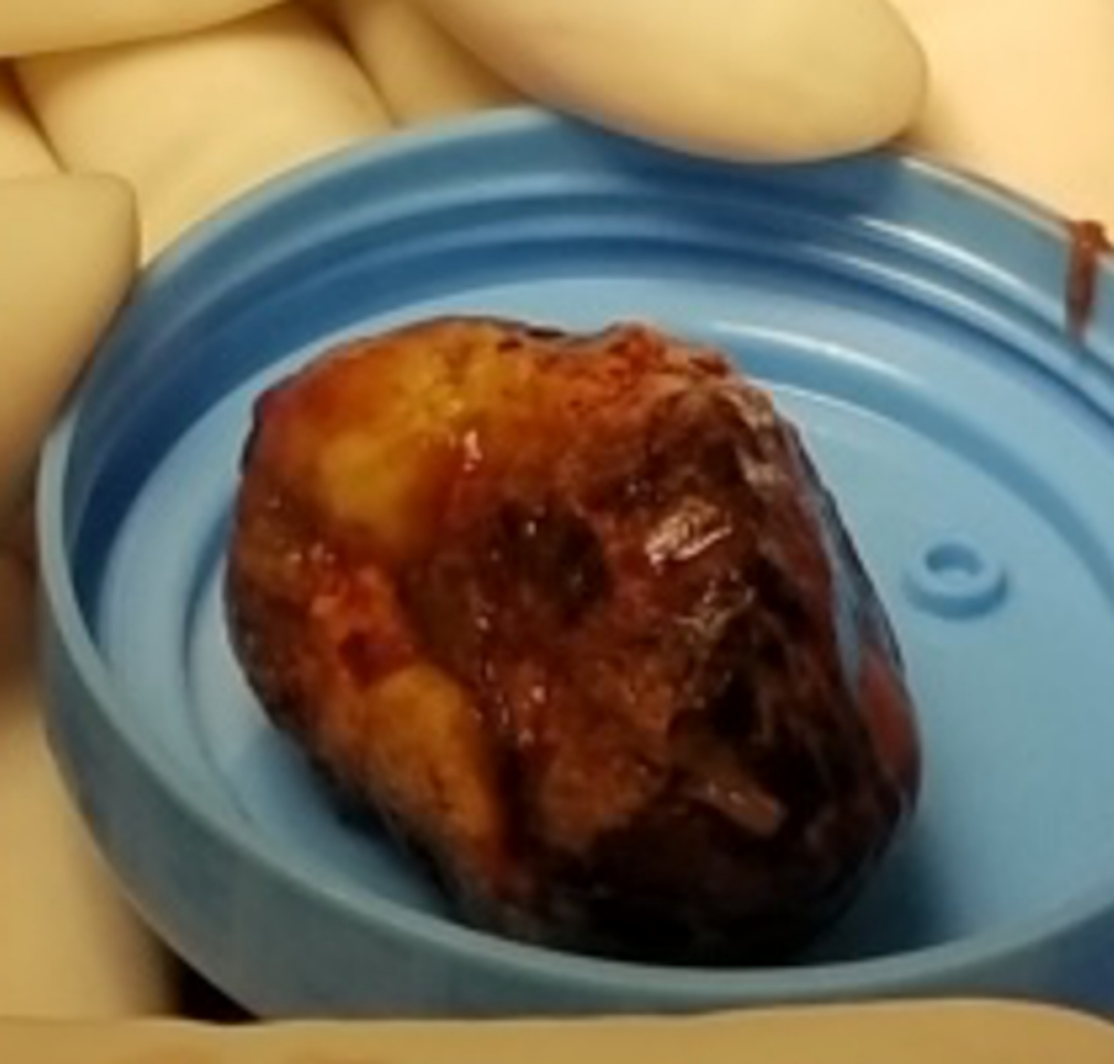
Large gallstone extracted from the small bowel

An 81-year-old female with a prior history of unresectable lung cancer status post radiation therapy, dementia, and multiple other comorbidities presented to the emergency room with acute onset nausea, vomiting, abdominal discomfort, and bloating. She was afebrile with vital signs significant for a blood pressure of 100/50 mmHg. Physical examination was significant for mild abdominal distention. Laboratory results were significant for AKI with a BUN of 81 mg/dL and a creatinine of 4.2 mg/dL (baseline 1.5 mg/dL). The patient underwent a CT scan of the abdomen that revealed pneumobilia and small bowel obstruction secondary to a radiolucent impacted gallstone in the distal ileum (Figure 4). The patient was fluid resuscitated over the next 24 hours and was subsequently taken for diagnostic laparoscopy. A supraumbilical video port was placed with two additional 5-mm ports placed in the left upper and left lower quadrants. The distal decompressed loops of small bowel were identified and run proximally to find a transition point (Figure 5). This was grasped and brought out through the midline mini laparotomy at the supraumbilical port (Figure 6). An enterotomy was performed and a large stone was extracted (Figure 7). Postoperatively, the patient was started on a diet on day one and discharged on postoperative day four.

## Discussion

The classic clinical presentation of gallstone ileus in an older woman with episodic subacute obstruction with nausea, vomiting, crampy abdominal pain, and/or variable distension is commonly present as was seen in both patients before surgical intervention [9]. The episodic obstruction is a result of the stone tumbling through the bowel lumen causing transient gallstone impaction resulting in diffuse abdominal pain and vomiting, which is relieved as the gallstone becomes disimpacted, and recurs again as the stone lodges in the more distal bowel lumen [2,9,10]. The mean symptom duration before hospital admission is approximately seven days and then an average of about four additional days in the hospital before surgical intervention [11]. On physical examination, the patient may be acutely ill and often appears dehydrated. Fever, toxicity and physical signs of peritonitis may be noted if perforation of the intestinal wall takes place. The physical exam may also be completely normal if no obstruction is present at the moment [12]. In addition, 20% of patients in one series had signs consistent with acute cholecystitis [13]. The patients presented did not have signs of peritonitis or significant laboratory results besides mild hemoconcentration due to dehydration, and a decreased GFR due to chronic kidney disease in the second patient. In the literature, laboratory studies are usually nonspecific and may include leukocytosis, electrolyte imbalance due to dehydration which must be corrected before surgery, and elevated aminotransferase levels [5].

Computed tomography (CT) is the preferred modality in comparison to plain abdominal films or ultrasound in the diagnosis of gallstone ileus with a sensitivity of up to 93% [14]. When diagnosing gallstone ileus on CT imaging, one study found two of the three following signs, known as Rigler’s triad, in about 78% of the cases as opposed to about 15% with X-ray [15]. Rigler's triad is the appearance of pneumobilia, small bowel obstruction, and gallstone (usually in the iliac fossa) [16]. In Bouveret’s syndrome, one can expect to see on CT obstruction due to a gastroduodenal mass, pericholecystic inflammatory changes extending into the duodenum, gas in the gallbladder, pneumobilia or cholecystoduodenal fistula, filling defects corresponding to gallstones, a thickened gallbladder wall, and/or a contracted gallbladder [17].

Multiple surgical approaches to relieve the obstruction have been defined in the literature, but, since this condition is so unusual, there has been no accepted consensus regarding its management. Our approach was a mini-laparotomy at the supraumbilical port for stone extraction, however, the current surgical approaches include: simple enterolithotomy as was performed; enterolithotomy, cholecystectomy and fistula closure (one-stage procedure); and enterolithotomy with cholecystectomy performed later (two-stage procedure) [18]. Enterolithotomy has been the most common surgical procedure performed through an exploratory laparotomy after the site of gastrointestinal obstruction is localized. A longitudinal incision is made on the antimesenteric border proximal to the site of gallstone impaction where, if the stone cannot be manipulated, it is removed through an enterotomy right above it for extraction [5,17]. Manipulation of stones through the cecum has been associated with mucosal injury and undetected serosal rupture and therefore should not be performed routinely [5]. Laparoscopic-guided enterolithotomy has been performed in selected cases, though not widespread as this approach can be challenging due to the difficulty of examining a dilated small bowel and identifying the gallstone through the laparoscope. If a laparoscopic approach is used, it is best to mobilize and identify the obstructed loop of the bowel first and then perform the stone extraction after eviscerating the loop of the bowel through a limited incision in order to minimize the spillage of enteral contents into the abdomen [19]. It was generally believed that a simple enterolithotomy was the safest approach to relieve symptoms in the immediate, but since the majority of patients with gallstone ileus are older adults and have other serious medical conditions, the morbidity and mortality rate for gallstone ileus remains high with a recent study of 127 patients reporting 35.4% and 5.5%, respectively [20]. While most authors have advocated for open surgery, however, in view of elderly age and multiple comorbidities as seen in the patients presented, a laparoscopic approach may provide faster recovery with minimal morbidity as both patients were discharged by day 4 with no readmission.

## Conclusions

Gallstone ileus is primarily a geriatric disease in patients with multiple comorbidities. Use of a laparoscope and a mini laparotomy to perform an enterolithotomy provides short operative time with minimal postoperative morbidity and early recovery.

## References

[1] Abou-Saif A, Al-Kawas FH (2002). Complications of gallstone disease: Mirizzi syndrome, cholecystocholedochal fistula, and gallstone ileus. Am J Gastroenterol.

[2] Fox PF (1970). Planning the operation for cholecystoenteric fistula with gallstone ileus. Surg Clin North Am.

[3] Despland M, Clavien PA, Mentha G, Rohner A (1992). Gallstone ileus and bowel perforation after endoscopic sphincterotomy. Am J Gastroenterol.

[4] Halabi WJ, Kang CY, Ketana N (2014). Surgery for gallstone ileus: a nationwide comparison of trends and outcomes. Ann Surg.

[5] Clavien PA, Richon J, Burgan S, Rohner A (1990). Gallstone ileus. Br J Surg.

[6] Nakao A, Okamoto Y, Sunami M, Fujita T, Tsuji T (2008). The oldest patient with gallstone ileus: report of a case and review of 176 cases in Japan. Kurume Med J.

[7] Yılmaz EM, Cartı EB, Kandemir A (2018). Rare cause of duodenal obstruction: Bouveret syndrome. Turk J Surg.

[8] Rodrigues ILM (2018). Bouveret syndrome and its imaging diagnosis. Radiol Bras.

[9] Masannat Y, Masannat Y, Shatnawei A (2006). Gallstone ileus: a review. Mt Sinai J Med.

[10] Warshaw AL, Bartlett MK (1966). Choice of operation for gallstone intestinal obstruction. Ann Surg.

[11] Cooperman AM, Dickson ER, ReMine WH (1968). Changing concepts in the surgical treatment of gallstone ileus: a review of 15 cases with emphasis on diagnosis and treatment. Ann Surg.

[12] VanLandingham SB, Broders CW (1982). Gallstone ileus. Surg Clin North Am.

[13] Moss JF, Bloom AD, Mesleh GF, Deziel D, Hopkins WM (1987). Gallstone ileus. Am Surg.

[14] Yu CY, Lin CC, Shyu RY (2005). Value of CT in the diagnosis and management of gallstone ileus. World J Gastroenterol.

[15] Lassandro F, Gagliardi N, Scuderi M, Pinto A, Gatta G, Mazzeo R (2004). Gallstone ileus analysis of radiological findings in 27 patients. Eur J Radiol.

[16] Koulaouzidis A, Moschos J (2007). Bouveret’s syndrome. Narrative review. Ann Hepatol.

[17] Kurtz RJ, Heimann TM, Beck AR, Kurtz AB (1985). Patterns of treatment of gallstone ileus over a 45-year period. Am J Gastroenterol.

[18] Nuño-Guzmán CM, Marín-Contreras ME, Figueroa-Sánchez M, Corona JL (2016). Gallstone ileus, clinical presentation, diagnostic and treatment approach. World J Gastrointest Surg.

[19] Behrens C, Amson B (2010). Laparoscopic management of multiple gallstone ileus. Surg Laparosc Endosc Percutan Tech.

[20] Mallipeddi MK, Pappas TN, Shapiro ML, Scarborough JE (2013). Gallstone ileus: revisiting surgical outcomes using National Surgical Quality Improvement Program data. J Surg Res.

